# Establishing an algorithm for molecular genetic diagnostics in Chinese children with brachydactyly type E

**DOI:** 10.3389/fendo.2025.1571136

**Published:** 2025-06-16

**Authors:** Xueqian Wang, Shengzhuang Guan, Yiqing Gao, Rongrong Xie, Fengyun Wang, Xiuli Chen, Haiying Wu, Xiaohui Zhang, Dandan Zhang, Bingyu Yang, Qisang Fan, Qing Wang, Hongying Wang, Tao Feng, Haitao Lv, Ting Chen

**Affiliations:** ^1^ Suzhou Clinical Center for Rare Diseases in Children, Children’s Hospital of Soochow University, Suzhou, Jiangsu, China; ^2^ Department of Endocrinology, Genetics and Metabolism, Children’s Hospital of Soochow University, Suzhou, Jiangsu, China; ^3^ Department of Endocrinology and Metabolism, Xuzhou Children’s Hospital, Xuzhou, Jiangsu, China; ^4^ State Key Laboratory of Common Mechanisms Research for Major Diseases, Suzhou Institute of Systems Medicine, Chinese Academy of Medical Sciences & Peking Union Medical College, Suzhou, China

**Keywords:** brachydactyly, genotype, phenotype, algorithm, children

## Abstract

**Background:**

Brachydactyly type E (BDE) is characterized by variable shortening of metacarpals or metatarsals, often involving phalanges. It may occur as an isolated anomaly or as part of congenital syndromes. With advancements in molecular diagnostic technologies, how genetic testing enhances the precise diagnosis of BDE remains unclear. Our aims were to establish an algorithm for molecular genetic diagnostics in Chinese children with BDE and to explore the phenotype-genotype correlations of Chinese patients with BDE.

**Methods:**

We reviewed left-hand wrist X-rays from children visiting Children’s Hospital of Soochow University (Jun 2021–Dec 2023). From 60,650 films, 135 BDE cases were identified, and their comprehensive phenotypes were collected. Whole-exome sequencing (WES) with copy number variation (CNV) analysis was performed on 60 patients and their parents. Sanger sequencing was used to validate single nucleotide variants (SNV) and indels.

**Results:**

Causative variants were found in 19 patients. SNVs and indels affecting 10 genes were identified in 15 patients, and CNVs in four. *GNAS* mutations were the leading cause (four cases), followed by *EXT1* and *ACAN* defects. The diagnostic yield was 19.1% in patients with isolated brachydactyly; 75% in patients with brachydactyly combined with short stature; 77.8% in patients with brachydactyly combined with facial dysmorphism; 83.3% in patients with brachydactyly combined with intellectual disability.

**Conclusion:**

Through comprehensive evaluation of genotype-phenotype correlations, we propose a diagnostic algorithm for precise molecular diagnosis in Chinese children with BDE.

## 1 Introduction

Brachydactyly (BD) refers to the shortening of the hands/feet caused by abnormal development of the metacarpals, metatarsals and/or phalanges. BD is classified into five types, A through E, based on anatomical characteristics (1). Most types of BD are rare, with the exception of types A3 (BDA3, OMIM 112700) and D (BDD, OMIM 113200) (2). Brachydactyly type E (BDE) involves variable shortening of the metacarpals/metatarsals, with frequent involvement of the phalanges. As a minor morphologic anomaly, BDE can occur in isolation or as a feature of various congenital syndromes (3, 4).

To date, mutations in two genes have been associated with isolated BDE. Brachydactyly type E1 (BDE1, OMIM 113300) is caused by a heterozygous mutation in the HOXD13 gene. Some patients with HOXD13 gene variants present not only with BDE, but also with overlapping features of BDD, characterized by the shortening and broadening of thumbs (5–7). Brachydactyly type E2 (BDE2, OMIM 613382) is caused by a heterozygous mutation in the *PTHLH* gene. Most, but not all, patients with *PTHLH* gene defect exibit short stature in addition to BDE (8–12).

The most common syndromic forms of BDE are inactivating PTH/PTHrP signaling disorders (iPPSD), such as pseudohypoparathyroidism 1A (PHP1A, OMIM 103580), pseudopseudohypoparathyroidism (PPHP, OMIM 612463), acrodysostosis with multihormonal resistance (ACRDYS1, OMIM 101800), acrodysostosis without multihormonal resistance (ACRDYS2, OMIM 614613), and hypertension with brachydacytly syndrome (HTNB, OMIM 112410) (13–15). Patients with these conditions often exhibit features of Albright Hereditary Osteodystrophy (AHO), which include BDE, short stature, obesity, round face, subcutaneous ossifications, and other skeletal anomalies (16–18). Syndromic BDE can also be related to chromosomal aberrations, with the most common disorder being Turner syndrome. BDE is also observed in 2q37 deletion syndrome, alternatively known as brachydactyly mental retardation syndrome (BDMR, OMIM 600430) (19, 20). The most common phenotypic features of BDMR comprise developmental delay, BDE, short stature, obesity, autistic features, and craniofacial or skeletal dysmorphism (21–23).

In this study, we reviewed the left-hand wrist X-ray film of 60,650 children and identified 135 children with BDE. Among these, 60 unrelated families consented to genetic testing. Our objectives are to establish an algorithm for molecular genetic diagnostics in Chinese children with BDE, and to explore the phenotype-genotype correlations of Chinese patients with BDE. This research may provide insights into the necessity of genetic investigations for BDE in future clinical practice.

## 2 Methods

### 2.1 Study design and participants

**Figure 1:**
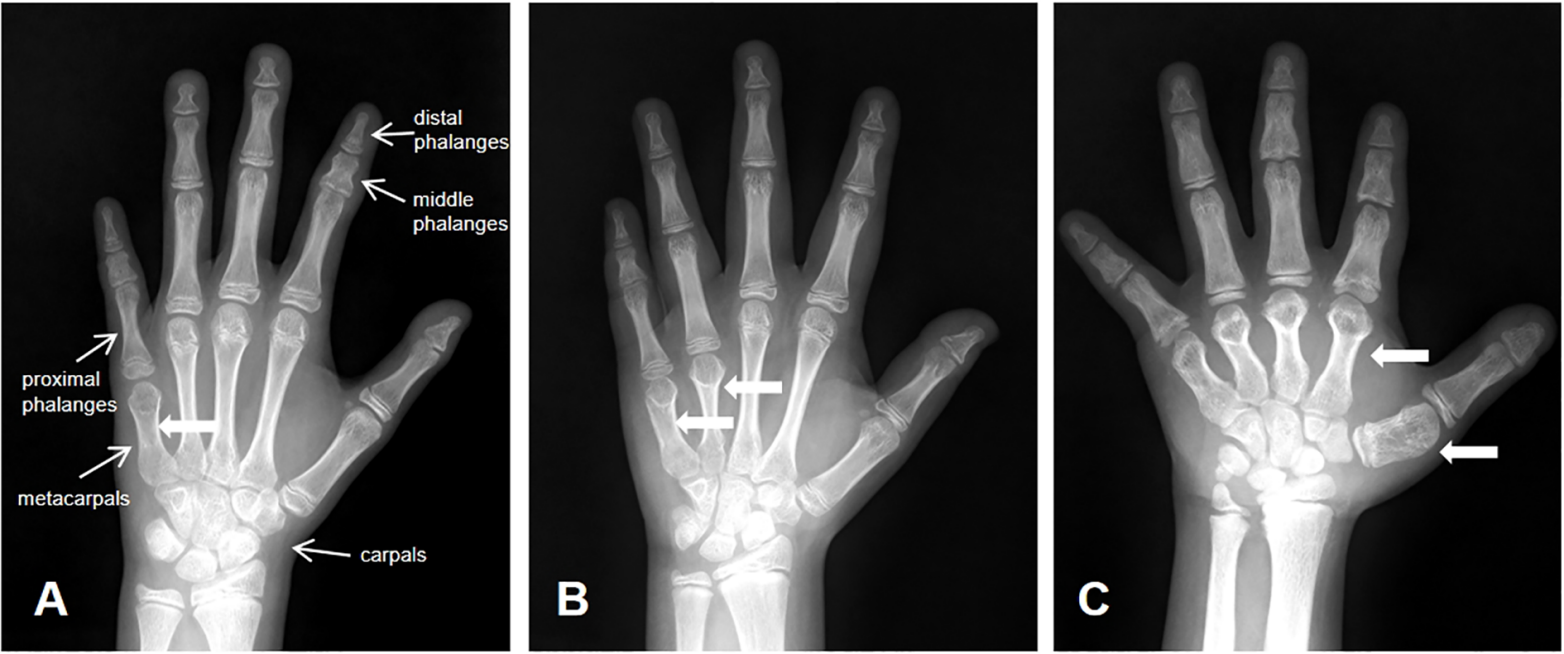
Diagnostic imaging. **(A)** BDE with shortened fifth metacarpal; **(B)** BDE with shortened fourth and fifth metacarpals; **(C)** BDE with shortened first to fifth metacarpls.

**Figure 2:**
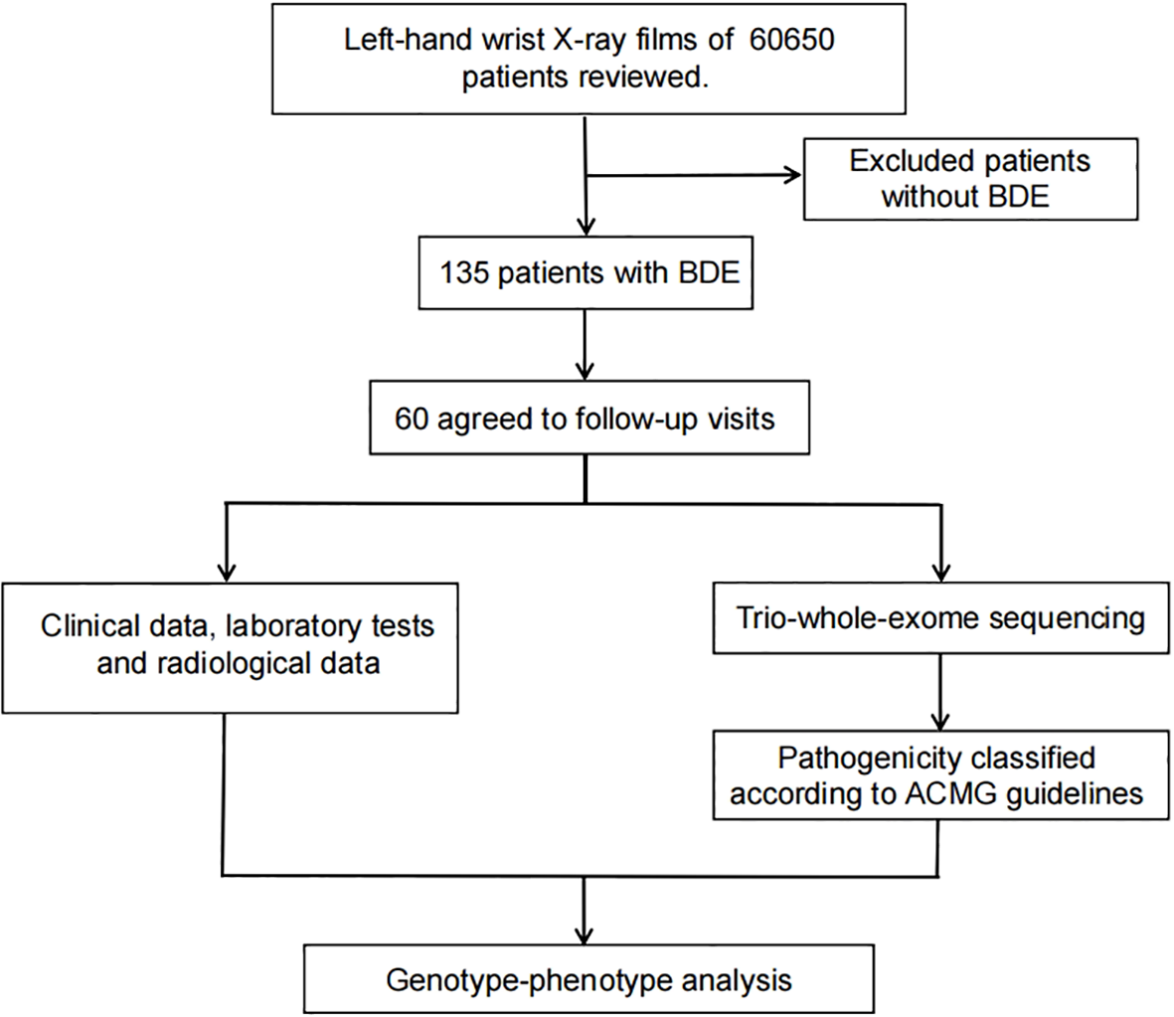
Flowchart depicting patient enrollment and follow-up process.

Our study retrospectively analyzed left-wrist radiographs from 60,650 children who did this test at Children’s Hospital of Soochow University between June 2021 and December 2023. **Figure 1** shows the diagnostic imaging. Out of 60,650 children, we identified 135 patients with BDE. With fully informed consent, 60 patients and their parents agreed to participate in follow-up visits and underwent Trio-whole-exome sequencing (Trio-WES) (**Figure 2**). This study received approval from the Ethics Committee of Children’s Hospital of Soochow University.

### 2.2 Data collection

We retrieved clinical data from the medical charts of 135 patients with BDE. Additionally, we collected detailed phenotypic information from the 60 children and their family members who agreed to follow-up visits. Height was measured using a wall-mounted stadiometer, and weight was measured using a digital floor scale. Each measurement was repeated three times, and the average values were calculated. Height standard deviation scores (SDS) were calculated based on the normal range for Chinese children (24, 25).

The left-hand wrist X-ray films were reviewed by two well-trained pediatric endocrinologists and one radiologist. Bone age was assessed by radiograph using the Tanner-Whitehouse III method (26). Advanced bone age was defined as bone age exceeding the chronological age by one year or more, while delayed bone age was defined as bone age behind the chronological age by one year or more.

### 2.3 Genetic testing

Peripheral blood samples were collected from the patients and their parents after obtaining written informed consent. Genomic DNA was extracted from these samples. WES was performed using the Illumina HiSeq 2500 platform (Illumina, San Diego, CA, USA) with an average sequencing depth 120X. The sequences were aligned to the reference human genome (hg19). Candidate variants were validated by Sanger sequencing, and their pathogenicity was classified following the guidelines of the American College of Medical Genetics and Genomics (ACMG) (27). For SNV classification, population data were obtained from gnomAD, literature-based variant information was sourced from HGMD Professional (HGMDpro), and REVEL was used as an in silico prediction tool for missense variants. For CNV classification, the DECIPHER website (https://www.deciphergenomics.org/) was used to identify genes involved in the CNV, and dosage sensitivity information was obtained from the ClinGen Dosage Sensitivity Map.

### 2.4 Statistical analysis

Statistical analyses were performed using SPSS Statistics version 26. Continuous variables are presented as mean ± standard deviation, while categorical variables are reported as frequencies and percentages. The chi-square test was used to assess intergroup differences for categorical variables. A p-value of < 0.05 was considered statistically significant. To evaluate the performance of genetic testing across different clinical symptom profiles, we calculated diagnostic yield, sensitivity, and specificity using a 2×2 contingency table, comparing genetic test results against clinical diagnostic criteria.

## 3 Results

### 3.1 Demographic and clinical features

We reviewed left-hand wrist X-ray films from 60,650 children (female to male ratio 1.2), and identified 135 children with BDE (female to male ratio 6.1). The overall prevalence of BDE was 0.22%, with a higher prevalence of 0.35% in females and a lower prevalence of 0.07% in males. The average chronological age and bone age of the 135 BDE patients were 9.4 ± 2.3 years and 9.9 ± 2.6 years, respectively. Among these patients, 31.1% exhibited advanced bone age, and 12.6% showed delayed bone age. Most BDE patients had near-normal height (height SDS between -1 and 0), while approximately 6.7% patients were of short stature (height SDS<-2). About 80.0% BDE patients had shortening of metacarpals limited to the fourth or fifth metacarpals, 14.8% had shortening involving both the fourth and fifth metacarpals, and 5.9% had shortening of all metacarpals. A further 7.4% of patients had shortening of metatarsals. About 4.4% had shortening of metatarsals limited to the fourth or fifth metatarsals, 3.0% had shortening involving both the fourth and fifth metatarsals. Additionally, 32.0% of patients also had a shorter middle phalanx of fifth finger, known as BDA3; 5.0% BDE patients had a shorter middle phalanx of both the second and fifth finger, known as brachydactyly type A4 (BDA4, OMIM 112800); and 10.0% had short and broad thumbs, known as BDD.

With fully informed consent, 60 patients and their parents were recruited. Of these 60 patients, 50 were females and 10 were males. The average age was 9.1 ± 2.2 years, and their average bone age was at 10.0 ± 2.5 years. Similar to the overall statistics of the 135 BDE patients, among these 60 patients, 31.7% presented with advanced bone age, and 6.7% showed delayed bone age. Most patients had near-normal stature, while 6.7% had short stature. In addition to the shorting of metacarpal/metatarsal, these patients exhibited other skeletal abnormalities: three patients (5.0%) had multiple exostoses, three patients (5.0%) had pectus carinatum, four patients (6.7%) had elbow valgus, and one patient (1.7%) had knee valgus. Ten patients (16.7%) had a family history of brachydactyly. Nine patients (15.0%) exhibited facial dysmorphism, and six patients (10.0%) had intellectual disability.

### 3.2 Genetic findings

We identified causative single nucleotide variants (SNV) or small indels from ten different genes in 15 patients (**Table 1**) (28). The defect of the *GNAS* gene is identified as the most prevalent genetic cause, observed in 26.7% of patients with BDE. Defects of *EXT1* and *ACAN* were both detected in two patients, respectively. Other genetic abnormalities include variants of *IHH*, *PRKAR1A*, *PTHLH*, *PRMT7*, *POGZ*, *NPR2*, and *FBXW11* genes.

**Table 1:** Pathogenic and likely pathogenic genetic variants.

ID	Gene	Inheritance	Transcript	cDNA variant	Protein variant	Type	Source	ACMG classification	Evidence for ACMG classification
1	*GNAS*	AD	NM_000516.7	c.563_566del	p.Asp189MetfsTer14	Frameshift	Maternal	Pathogenic	PVS1+PM2_P
2	*IHH*	AD	NM_002181.4	c.1A>G	p.Met1?	Initial codon	Paternal	Likely pathogenic	PVS1_S+PM2_P+PS4_P
5	*PRKAR1A*	AD	NM_002734.5	c.982G>A	p.Ala328Thr	Missense	*De novo*	Likely pathogenic	PS2_M+PM2_P+PP3_M+PP2
7	*PTHLH*	AD	NM_198965.2	c.166C>T	p.Arg56Ter	Nonsense	*De novo*	Pathogenic	PVS1+PS2+PS4+PM2_P
8	*PRMT7*	AR	NM_019023.5	c.1168C>T	p.Arg390Ter	Nonsense	Maternal	Pathogenic	PVS1+PM2_P+PP4
				c.1283C>G	p.Thr428Arg	Missense	Paternal	Likely pathogenic	PM3+PM2_P+PP4+PP3
11	*GNAS*	AD	NM_000516.7	c.1006C>T	p.Arg336Trp	Missense	*De novo*	Likely pathogenic	PM6+PS4_M+PS3_P+PM2_P
15	*GNAS*	AD	NM_000516.7	c.136_138dup	p.Leu46dup	Duplication	*De novo*	Likely pathogenic	PM2_P+PM4+PM6_S+PP4+PS4_M
16	*ACAN*	AD	NM_001369268.1	c.2760_2761del	p.Asp922Ter	Nonsense	Paternal	Likely pathogenic	PVS1+PM2_P
17	*POGZ*	AD	NM_015100.4	c.1463del	p.Pro488LeufsTer16	Frameshift	*De novo*	Pathogenic	PVS1+PS2_P+PM2_P
25	*NPR2*	AR	NM_003995.4	c.1849delT	p.Trp617GlyfsTer101	Frameshift	Paternal	Likely pathogenic	PVS1+PM2_P
				c.1552T>C	p.Ser518Pro	Missense	Maternal	Variant of uncertain significance	PM3+PM2_P
27	*ACAN*	AD	NM_001369268.1	c.1130G>A	p.Trp377Ter	Nonsense	Maternal	Likely pathogenic	PVS1+PM2_P
31	*EXT1*	AD	NM_000127.3	c.1019G>A	p.Arg340His	Missense	*De novo*	Pathogenic	PS2+PS4+PM2_P+PM5+PP1+PP3
38	*FBXW11*	AD	NM_001378974.1	c.856T>C	p.Trp286Arg	Missense	*De novo*	Likely pathogenic	PM6+PM2_P+PP3_M+PP2
47	*GNAS*	AD	NM_000516.7	c.1174G>A	p.Glu392Lys	Missense	Maternal	Likely pathogenic	PS4+PM2_P+PP3_M
48	*EXT1*	AD	NM_000127.3	c.1154T>A	p.Leu385Ter	Nonsense	Maternal	Pathogenic	PVS1+PP4+PM2_P

AD, autosomal dominance; AR, autosomal recessive; _P;surpporting _M:Moderate _S:Strong.

Pathogenic copy number variations (CNV) were identified in four patients (**Table 2**) (28). Patient 12 exhibited a duplication on 16q22.3-24.3, encompassing 132 protein coding genes. Patient 35 presented with a deletion on 7q11.21-11.22, including the *AUTS2* gene, which overlaps with Mental Retardation, Autosomal Dominant 26 (MRD26, OMIM 615834). Patient 44 had a deletion on 2q37.2-37.3 containing *HDAC4* gene, known to be chromosome 2q37 deletion syndrome (2q37DS, OMIM 600430). Lastly, patient 56 was found to have a deletion of approximately 950kb on chromosome 17, affecting the *NF1* and adjacent genes, which is known as NF1 microdeletion syndrome (OMIM 613675).

**Table 2:** Pathogenic and likely pathogenic CNVs.

ID	Genomic coordinates (GRCh37)	Size(kb)	OMIM gene (n)	Source	ACMG classification	Evidence for ACMG classification
12	chr16:g.73437091-90155062dup	16718	127	*De novo*	Pathogenic	3C+4E
35	chr7:g.65996757-70598046del	4601	6	*De novo*	Pathogenic	2A+4C
44	chr2:g.236715862-242815502del	6100	51	*De novo*	Pathogenic	2A+3C+4C
56	chr17:g.29422387-30380347del	950	8	*De novo*	Pathogenic	2A+4A

### 3.3 Genotype-phenotype correlations

The comparison of clinical characteristics between genetically positive cases and negative cases was depicted in **Table 3**. The most recurrent phenotype among positive cases was two or more shortened metacarpals/metatarsals (n=15, 78.9%). Most patients had near-normal stature, while four patients (6.7%) had short stature, within whom three carried genetic abnormalities. Nine patients (15.0%) exhibited facial dysmorphism, within whom seven carried genetic abnormalities. Six patients (10.0%) had intellectual disability, and five of them carried genetic abnormalities. Clinical characteristics of 19 patients carrying causative variants are shown in **Table 4**.

**Table 3:** Summary of the clinical characteristics of the 135 patients.

Characteristic	Total (N=135)	Genetic testing cases (N=60)	Positive cases (N=19)	Negative cases (N=41)	P
Sex					0.082
Male	19	10	6(60%)	4(40%)	
Female	116	50	13(26%)	37(74%)	
Bone age					
Normal	76	37	10(27%)	27(73%)	0.605
Advanced	42	19	7(36.8%)	12(63.2%)	
Delayed	17	4	2(50%)	2(50%)	
Height SDS					0.035
>0	56	25	4(16.0%)	21(84.0%)	
between -1 and 0	44	17	5(29.4%)	12(70.6%)	
between -2 and -1	26	14	7(20%)	7(50%)	
<-2	9	4	3(75%)	1(25%)	
Number of shortened metacarpals/metatarsals					0.0001
1	100	36	4(11.1%)	32(88.9%)	
2	23	13	6(46.2%)	7(53.8%)	
>2	12	11	9(81.8%)	2(18.2%)	
Family history of brachydactyly					0.804
With	11	10	4(40%)	6(60%)	
Without	83	50	15(30%)	35(70%)	
NA	41	–	–	–	
Facial dysmorphism					0.005
With	10	9	7(77.8%)	2(22.2%)	
Without	84	51	12(23.5%)	39(76.5%)	
NA	41	–	–	–	
Intellectual disability					0.016
With	6	6	5(83.3%)	1(16.7%)	
Without	88	54	14(25.9%)	40(74.1%)	
NA	41	–	–	–	

SDS, standard deviation score.

**Table 4:** Clinical characteristics of 19 patients with pathogenic/likely pathogenic variants.

ID	Sex	Age (years)	BA (years)	Height (cm)	Height SDS	MPH SDS	Skeletal anomalies	Other systemic abnormalities	Diagnosis
1	F	7.2	9.0	121.4	-0.4	-0.7	Short 1st to 5th metacarpals	ID	PHP1A
2	F	8.7	11.8	141.1	1.6	-1	Short 5th metacarpals	None	ACFD
5	F	5.3	6.3	102.1	-2.4	-1	Short 4th and 5th metacarpals	None	ACRDYS1
7	F	10.3	11.3	140.5	-0.3	1.1	Short 4th and 5th metacarpals	None	BDE2
8	F	10.1	7.1	118.6	-3.5	-0.2	Short 1st to 5th metacarpals	ID, dysmorphic facial features (malar hypoplasia, depressed nasal bridge, abnormal occlusion of teeth), seizures	SBIDDS
11	M	7.9	9.1	122.5	-1.3	-0.8	Short 4th and 5th metacarpals, 4th and 5th metatarsals	None	PHP1A
12	F	11.2	11	140.3	-1.1	-0.1	Short 5th metacarpals, 4th and 5th metatarsals, early closure of the epiphyses, elbow valgus, knee valgus	ID, dysmorphic facial features (high and narrow forehead, low and flat cheekbones, uneven teeth, wide and flat philtrum), PDA	NA
15	M	10.4	14.5	148.9	1.1	-1.2	Short 1st to 5th metacarpals, second metatarsals	None	PHP1A
16	M	8.9	9.0	124.5	-1.8	-0.9	Short 1st to 5th metacarpals	None	SSOAOD
17	F	8.7	10.9	139.5	1.3	0.4	Short 4th metacarpals	ID, dysmorphic facial features (strabismus, malar hypoplasia, depressed nasal bridge, pointed chin)	WHSUS
25	M	5.7	6.1	103.2	-2.8	-0.9	Short 1st to 5th metacarpals	None	AMDM
27	F	11.8	11.0	139.3	-1.8	-1	Short 4th and 5th metacarpals	None	SSOAOD
31	M	13.3	14.1	148.3	-1.7	-0.6	Short 4th and 5th metacarpals, exostoses, pectus carinatum	None	MHE
35	F	11.5	11.7	138.5	-1.7	-0.6	Short 5th metacarpals	Dysmorphic facial features (broad forehead, thick lips)	MRD26
38	M	7.8	10.3	123.7	-0.9	0.1	Short 5th metacarpals	Dysmorphic facial features (frontal bossing, prognathism)	NEDJED
44	F	9	9.2	136.8	0.5	2.2	Short 1st to 5th metacarpals, pectus carinatum	ID, dysmorphic facial features (hypertelorism, strabismus, micrognathia), ADHD	2q37DS
47	F	5.8	6.7	106.7	-2	-1	Short 4th and 5th metacarpals	None	PHP1A
48	F	12.6	14.7	150.3	-0.7	-2.7	Short 4th metacarpals, 4th metatarsals, exostoses	None	MHE
56	F	13.2	12	152	-0.8	0.9	Short 4th and 5th metacarpals, 4th and 5th metatarsals, polydactyly of the left foot	Dysmorphic facial features (sparse eyebrows, small eye slits, downward slanting outer eyelids)	NF1 microdeletion syndrome

F, female; M, male; BA, bone age; SDS, standard deviation score; MPH, midparental height; NA, not available; ID, intellectual disability; PDA, patent ductus arteriosus;ADHD, attention deficit hyperactivity disorder; PHP1A, pseudohypoparathyroidism 1A; ACFD, acrocapitofemoral dysplasia; ACRDYS1, Acrodysostosis-1; BDE2, brachydactyly type E2; SBIDDS, short stature, brachydactyly, impaired intellectual development, and seizures; SSOAOD, short stature and advanced bone age with or without early-onset osteoarthritis and/or osteochondritis dissecans; WHSUS, White-Sutton syndrome; AMDM, Acromesomelic dysplasia-1; MHE, multiple hereditary exostoses; MRD26,Mental Retardation, Autosomal Dominant 26; NEDJED, neurodevelopmental, jaw, eye, and digital syndrome; 2q37DS, chromosome 2q37 deletion syndrome.

Patients with shorter stature were significantly more likely to have genetic abnormalities (P=0.035). The number of shortened metacarpals and metatarsals was notably higher in positive cases compared to negative cases (p=0.0001). Additionally, the frequency of dysmorphic facial features was significantly greater in positive cases than in negative cases (P=0.005). Intellectual disability was also more frequent in positive cases, with a statistically significant difference (P=0.016).

### 3.4 Diagnostic yield in patients with different phenotypes

In patients with isolated brachydactyly, the diagnostic yield was 19.1%, sensitivity was 47.4%, and specificity was 7.3%. In patients with additional clinical manifestations, the diagnosis yield, sensitivity, and specificity increased, as shown in **Table 5**. Among them, the highest diagnosis yield of 83.3% was observed in patients with intellectual disability; the highest sensitivity of 78.9% was seen in patients with multiple metacarpal/metatarsal shortening; and the highest specificity of 97.6% was found in patients with short stature and intellectual disability.

**Table 5:** Performance of the prediction rule for diagnosing brachydactyly.

Clinical Characteristics	Diagnostic yield (%)	Sensitivity (%)	Specificity (%)
Isolated brachydactyly	19.1	47.4	7.3
Brachydactyly combined with short stature	75	15.8	97.6
Brachydactyly combined with facial dysmorphism	77.8	36.8	95.1
Brachydactyly combined with intellectual disability	83.3	26.3	97.6
Multiple metacarpal/metatarsal shortening	62.5	78.9	78

## 4 Discussion

The aim of the present study was to investigate the prevalence of BDE and to assess the necessity of genetic testing for BDE within a pediatric medical center setting. We reviewed left-hand wrist X-ray films from 60650 children and identified 135 cases with BDE, which was more common in females than in males. Genetic testing was performed on 60 patients, revealing identified genetic etiology in 31.7% of them. In total, 15 variants from ten genes and four CNVs were identified in 19 patients. The genetic diagnostic yield was significantly higher in patients presenting with short stature, multiple shortened metacarpals or metatarsals, facial dysmorphism, and intellectual disability compared to those without these phenotypes.

The pronounced female predominance in our cohort (female-to-male ratio 6:1) significantly exceeds both our screened population baseline (1.2:1) and general population expectations. While BDE gender ratios remain unreported, our findings align with documented female predominance in subtypes of brachydactyly type A (BDA). A study in Chinese children found that out of 174 patients with BDA3, 111 were females. Even after excluding females with Turner syndrome, the prevalence of BDA3 in females remained significantly higher than in males (29). Similarly, surveys conducted in Native Americans, Mexico, and Pacific islander populations have also reported a higher frequency of BDA3 in females (30, 31). This disparity suggests either ethnic-specific biological factors, estrogen-mediated modulation of skeletal development pathways, or influence by genes on X-chromosome. To further validate this observation, we recommend collaborative meta-analyses of gender-stratified data from multinational consortia to distinguish true biological sex differences from sampling artifacts.

In this study, we found that the genetic etiology of BDE can be roughly divided into three categories. Approximately one-third of BDE patients have defects in PTH/PTHrP signaling (13–15), with *GNAS* gene defects being the most common within this group. Patients with iPPSDs, including PHP1A, PHP1C, ACRDYS1 and ACRDYS2, commonly present with BDE. Another one-third of BDE patients exhibit defects in growth plate or cartilage signaling, which include deficiencies in genes such as *ACAN*, *IHH* and *EXT1*. Defects in these genes typically result in short stature, although the presence of BDE is not consistent, with variations observed even among family members carrying the same mutation. The last one-third of patients display diverse genetic profiles, often accompanied by neurological manifestations in addition to BDE. Notably, patient 8 was found to have biallelic variants in the *PRMT7* gene, causing short stature, brachydactyly, intellectual disability, dysmorphic facial features, and seizures syndrome (SBIDDS) (32–34), which aligns with her clinical presentation. Patient 17 had a *de novo* frameshift mutation in the *POGZ* gene, which is associated with White-Sutton syndrome (WHSUS, OMIM 616364) (35, 36). WHSUS is a neurodevelopmental disorder characterized by a spectrum of intellectual disabilities, global developmental delay and behavioral problems, short stature, dysmorphic facial features and brachydactyly (37–40).

In the present study, approximately one-third of patients who underwent genetic testing were found to have a genetic cause for their condition. Notably, individuals with isolated BDE, without accompanying symptoms did not exhibit detectable genetic defects. The key risk factors for identifying a genetic etiology in BED include the severity of short stature, multiple shortened metacarpals/metatarsals, the extent of facial dysmorphism, and the presence of intellectual disability.

We describe three instructive cases highlighting the genetic complexity of BDE. Patient 12 harbored a rare 16q22.3-24.3 duplication, exhibiting intellectual disability, patent ductus arteriosus (PDA), and skeletal anomalies including BDE—expanding the phenotypic spectrum of distal 16q duplications beyond the six previously reported cases with the duplicated region spanning from 6Mb to 17Mb (41–44). Patient 35 carried a deletion in the chromosomal region 7q11.21-11.22, which includes the *AUTS2* gene, a key regulator of neuronal gene expression. This deletion is associated with MRD26 (45). Patient 35 exhibited BDE and short stature but normal cognition, challenging the classic MRD26 paradigm (46, 47). Patient 56 had a deletion of approximately 950kb on chromosome 17. This deletion, affecting *NF1* and contiguous genes, is associated with NF1 microdeletion syndrome (48–50). Besides common symptoms of NF1, patient 56 exhibited BDE and polydactyly, reinforcing the skeletal involvement in NF1 microdeletion syndrome (51, 52).

**Figure 3:**
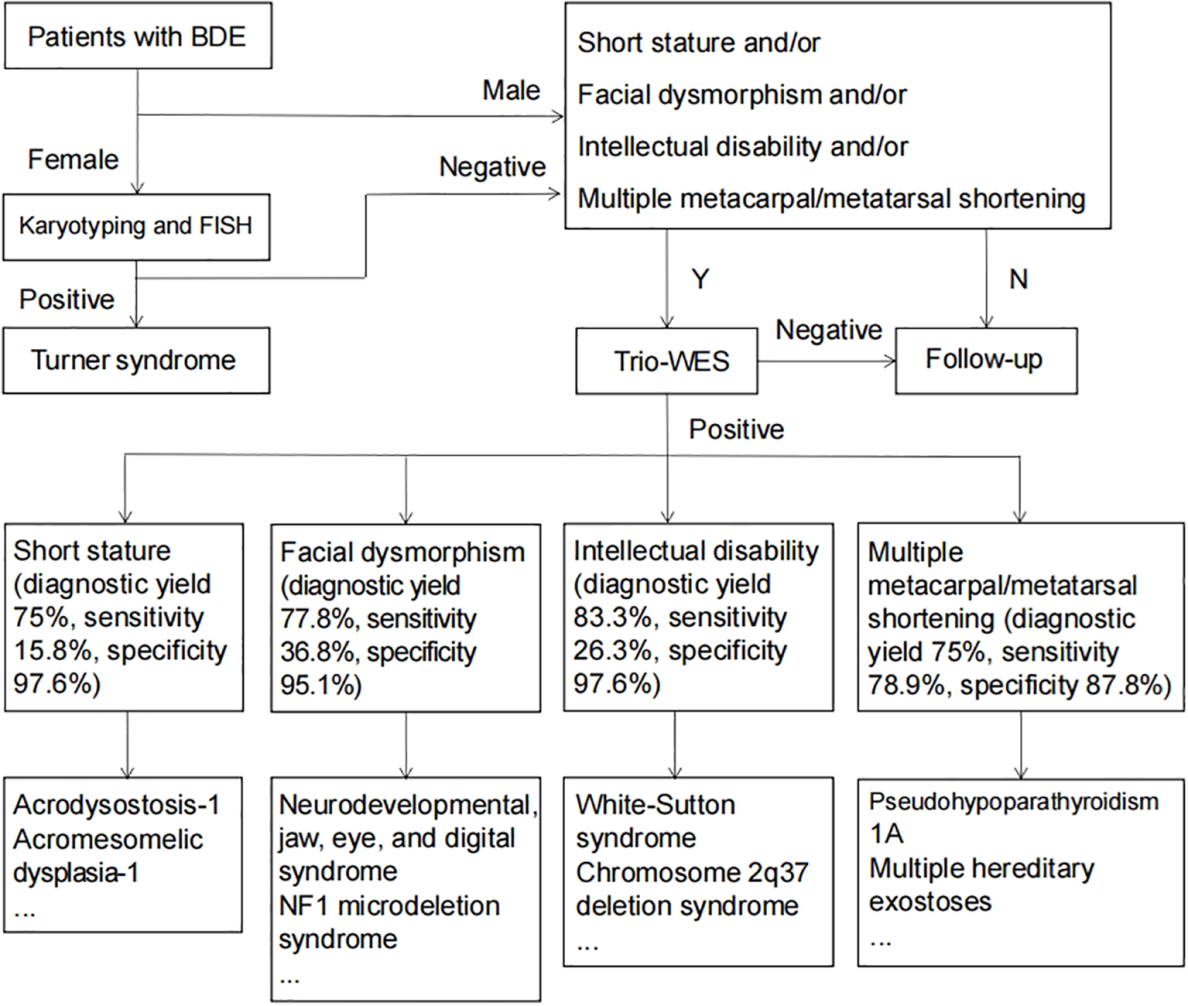
Diagnostic flowchart for patients with BDE.

Our comprehensive evaluation of 60 brachydactyly type E (BDE) patients reveals crucial insights into the diagnostic approach for this condition. In cases of isolated BDE, genetic testing demonstrates limited clinical utility. Routine molecular testing may not be justified for patients presenting with isolated digital shortening without additional clinical features. However, our data demonstrate dramatic improvements in diagnostic accuracy when BDE is accompanied by specific phenotypic red flags. The presence of even one additional clinical feature - particularly short stature (75% diagnostic yield), facial dysmorphism (77.8%), intellectual disability (83.3%), or multiple shortened metacarpals/metatarsals (62.5%) - increases the likelihood of identifying a genetic etiology by 3–4 fold compared to isolated cases. These clinical features serve as powerful predictors of underlying genetic pathology and should trigger more comprehensive evaluation. Based on these findings, we developed a phenotype-driven diagnostic algorithm (**Figure 3**) that optimizes testing efficiency while minimizing unnecessary procedures. In pediatric endocrinology practice, clinicians evaluating children with growth disorders should maintain particular vigilance for BDE, especially when assessing patients presenting with short stature, facial dysmorphism, or intellectual disability. A systematic examination of digital morphology should be performed, with particular attention to characteristic features such as shortened fourth/fifth metacarpals and positive Archibald’s sign. Upon identifying suspicious findings, clinicians are advised to implement our proposed diagnostic cascade beginning with standardized anthropometric documentation, followed by radiographic bone age assessment incorporating metacarpal pattern analysis, and culminating in targeted genetic testing.

While our study reveals significant genotype-phenotype correlations in BDE, several limitations should be noted. First, our selection approach of left-wrist radiographs may introduce bias toward children with growth abnormalities, potentially affecting prevalence estimates for incidental BDE findings. However, the large sample size helps mitigate this limitation for assessing overall BDE characteristics. Secondly, only a subset of BDE families consented to comprehensive genetic testing, resulting in a small sample size that may limit the generalizability of our findings and reduce statistical power for calculations. Besides, our study did not account for potential confounding factors such as epigenetic regulation, environmental influences, or genetic modifiers, which may contribute to phenotypic heterogeneity, especially for isolated brachydactyly. Furthermore, the pathogenic mechanisms of rare variants and the functional implications of variants of uncertain significance (VUS) remain unresolved. Lastly, while our study provides comprehensive data on left-wrist radiographs, cases of BDE with purely isolated right-hand involvement may have been underrepresented. These gaps highlight the need for larger, multicenter studies incorporating functional assays and population-level analyses to validate our findings and clarify disease mechanisms.

## 5 Conclusion

In conclusion, we conducted a comprehensive clinical and genetic analysis of Chinese patients with BDE. Our findings indicate that BDE, while considered a minor anomaly, is not uncommon among Chinese children and demonstrates a higher prevalence in females compared to males. The study underscores the importance of recognizing key phenotypic risk factors—such as pronounced short stature, involvement of multiple shortened metacarpals/metatarsals, distinct facial dysmorphism, and intellectual disability—for identifying an underlying genetic etiology. These insights provide a foundation for targeted genetic evaluations, which can enhance diagnostic accuracy and inform clinical management strategies for patients with BDE.

## Data Availability

The data presented in the study are deposited in the clinvar database https://www.ncbi.nlm.nih.gov/clinvar/, accession numbers: SCV005328512, SCV005328514, SCV005328517, and SCV005328539.
