# Supplementary material for: Establishing an algorithm for molecular genetic diagnostics in Chinese children with brachydactyly type E
Source: Front Endocrinol (Lausanne). 2025 Jun 16;16:1571136. doi: 10.3389/fendo.2025.1571136 (PMC12206630; doi:10.3389/fendo.2025.1571136)
Supplement: Supplementary file 1 [file Table1.pdf]

Table S1: Radiographs and Sanger sequencing validation of variant carriers		
ID	Radiographs	Sanger sequencing validation
1	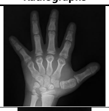	
2	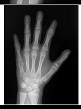	
5	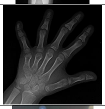	
7	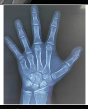	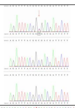
8	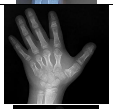	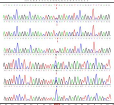
11	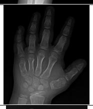	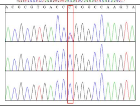
12	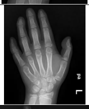	
15	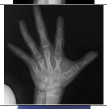	
16	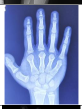	
17	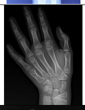	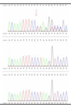
25	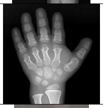	
27	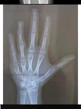	
31	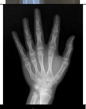	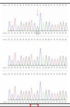
35	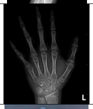	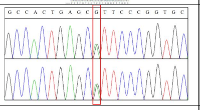
38	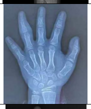	
44	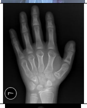	
47	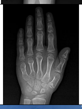	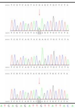
48	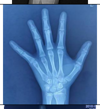	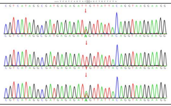
56	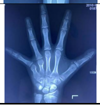	
